# Association between occupation and knee and hip replacement due to osteoarthritis: a case-control study

**DOI:** 10.1186/ar3033

**Published:** 2010-05-24

**Authors:** Jonas Franklin, Thorvaldur Ingvarsson, Martin Englund, Stefan Lohmander

**Affiliations:** 1 Department of Orthopedics, University Hospital, Eyrarlandsvegi, Akureyri, IS-600, Iceland; 2 Department of Orthopedics, Clinical Sciences Lund, Lund University, Lund, SE-22185, Sweden; 3 Department of Health Sciences, University of Akureyri, Nordurslod 2, IS-600, Iceland; 4 Faculty of Medicine, University of Iceland, Vatnsmyrarvegi 16, Reykjavík, IS-101, Iceland; 5 Clinical Epidemiology Research & Training Unit, Boston University School of Medicine, 650 Albany St., Suite X200, Boston, MA 02118, USA

## Abstract

**Introduction:**

The objective of this study was to examine the association between occupation and osteoarthritis (OA) leading to total knee (TKR) or hip (THR) joint replacement.

**Methods:**

The following is the case-control study design. All patients still living in Iceland who had had a TKR or THR due to OA as of the end of 2002 were invited to participate. First degree relatives of participating patients served as controls. N = 1,408 cases (832 women) and n = 1,082 controls (592 women), 60 years or older and who had adequately answered a questionnaire were analyzed. Occupations were classified according to international standards. Inheritance of occupations was calculated by using the Icelandic Genealogy Database.

**Results:**

The age adjusted odds ratio (OR) for male farmers getting a TKR due to OA was 5.1 (95% confidence interval (CI) 2.1 to 12.4) and for a male farmer getting a THR due to OA the OR was 3.6 (95% CI 2.1 to 6.2). The OR for a fisherman getting a TKR was 3.3 (95% CI 1.3 to 8.4). No other occupations showed increased risk for men. For women there was no increased risk for any occupation. Farming and fishing were also the occupations that showed the greatest degree of inheritance.

**Conclusions:**

These results support an association in males between occupations with heavy physical load and both TKR and THR for OA.

## Introduction

There are several possible risk factors for osteoarthritis (OA) of the knee and hip. Body mass index (BMI) is well established as a risk factor for both hip and knee OA for both genders [1,2]. It has been shown that high BMI precedes knee OA and thus causality can be assumed [3,4]. Occupation is another risk factor that has been linked to OA. Studies have varied in their design, in both definition of OA and exposure to workload. Most of the higher quality studies have based their definition on radiographic OA [5-9] or the case population has been individuals that are waiting for [10,11] or have received [12-17] a joint prosthesis. From a clinical standpoint, studies based on radiographic OA are less relevant, as there is poor correlation between radiographic OA and symptoms [18,19]. Exposure has either been defined from job titles [7,8,13,17] or exposure to certain tasks, estimated from job titles [5,20] or retrospectively by interviewing participants [6,11,14-16,21]. These methods all have their shortcomings: questionnaires are subject to recall bias but enable a more specific definition of the exposure (for example, lifting, kneeling), job titles are less prone to recall bias but give a more imprecise definition of the exposure and confounders that might be associated with certain professions. It has been established that farmers have an increased risk for hip OA [7-9,13,22,23], but few quality studies on knee OA have been based on job titles [12]. Recent reviews have concluded that there is moderate to strong evidence for heavy lifting and for farming as a risk factor for hip OA [24-26]. Similarly, it has been concluded that there is moderate evidence for a relationship between kneeling and heavy lifting and knee OA [26,27]. The majority of published studies have dealt either with hip or knee OA, relatively few studies have included both in the same cohort, thus enabling comparison of the risk for hip and knee OA. Most studies have been cross-sectional, but recently results from longitudinal studies have been published, strengthening the causality of occupation as a risk factor for knee [28] and hip OA [23,29].

The purpose of the present study was to explore the relationship between occupation and knee and hip OA leading to arthroplasty in Icelandic men and women. This case-control study compares 1,408 individuals with knee and or hip arthroplasty due to OA with their relatives of the same age that did not have total knee arthroplasty (TKR) or total hip arthroplasty (THR). The study subjects were recruited from all living TKR and THR cases in Iceland. The Icelandic healthcare system is publicly funded, thus minimizing bias due to socioeconomic status. The primary controls were all relatives to the cases, thus coming from the same genetic pool. Furthermore, we explored the inheritance of different professions within the cohort.

## Materials and methods

The study was approved by the Ethics Committee of Akureyri Central Hospital, Iceland, where the study was based.

This study is a part of a larger study on the effects of genes and environment on the risk for OA in the Icelandic population. The cases in that study were drawn from the entire Icelandic population representing all patients that had received a TKR or THR due to OA, and were alive and living in Iceland in 1998. In a clinical setting an orthopedic surgeon had decided that the severity of the patients' knee or hip OA warranted prosthetic surgery. In three of the six hospitals that have performed THR in Iceland, one of the authors checked all patient records to verify the correct diagnosis. If x-ray films were available, they were reviewed to confirm the diagnosis. Errors in the computer database diagnosis were found to be less than 2% [30]. We identified all 4,215 patients with a diagnosis of OA that had been operated on with TKR and (or) THR from the time of introduction of TKR and THR surgery in Iceland in 1967 until the end of year 2002 [30,31]. By cross referencing with the National Census and Death Register we identified 1,289 of these patients as deceased at the study start in 1998. A further 384 patients identified as living at the start of our study died before they were invited to participate. Patients that had moved abroad (n = 4) have only the country of residence registered and were therefore not traceable.

A total of 2,538 eligible cases were thus identified, of which 1,843 were accepted to participate in the main study (72.6%). From this study cohort we included those that met the following criteria: a verified diagnosis of OA, over 60 years of age at the time of surgery and having adequately answered the study questionnaire. This left us with 1,408 participating index cases of which 576 were men and 832 were women. All data were collected retrospectively after surgery.

One of the objectives of the main study was to search for genes associated with OA. To facilitate this search we used first degree relatives of cases as controls. All cases were asked to supply information about all their first degree relatives (parents, siblings and children) and these were contacted and asked to participate in the study as controls. Similar to the cases, we included only relatives who were 60 years of age or older at study entry and who had completed the questionnaire, leaving 490 men and 592 women as our controls. The cases and controls were thus of similar age and from the same genetic pool.

Cases and controls signed an informed consent and answered a questionnaire. The questionnaire contained 79 questions about the patient's height and weight, general health, occupation, family history, physical activities, and detailed description of all musculoskeletal symptoms, including duration of symptoms.

Participants were asked to grade their recreational physical activity as none, light, regular or heavy.

The occupations of cases and controls were classified according to the Icelandic translation of the ISCO-88 (International Standard Classification of Occupations 1988) published by The International Labour Organization (ILO) [32]. Several occupations had only a handful of individuals. We therefore categorized the occupation of our study subjects into eight groups (Table 1). Study participants reported all occupations they had had until study entry. The occupation held longest was registered as the occupation for that participant.

**Table 1:** Classification of occupations

ISCO-88 Class	Our class	Most common occupations for men	Most common occupation for women
Legislators, senior officials and managers	Managers and professionals	Teachers, doctors	Teachers, nurses
Professionals			
Technicians and associate professionals	Technicians and clerks	Office clerks, ship's engineers	Office clerks, nurses assistants
Office clerks			
Service workers and shop and market sales workers	Service and shop workers	Salespersons, police officers	Salespersons, catering personnel
Skilled agricultural and fishery workers	Farmers	Farmers	Farmers
	Fishermen	Fishermen	(none in group)
Craft and related trades workers	Craft workers	Carpenters, construction workers	Fish processing, sewers
Plant and machine operators and assemblers	Operators and unskilled labour	Building construction labourers, heavy truck- and lorry drivers	Cleaners, factory work
Elementary occupations			
Armed forces	Not applicable*		
	Housewives	(none in group)	Housewives

* There are no armed forces in Iceland.

We did separate analyses for men and women and for TKR and THR. Statistical comparisons between groups were done using the independent samples T test. We calculated odds ratios (OR) using logistic regression in a model adjusted for age, body mass index (BMI), and recreational physical activity. All analyses were carried out at the level of the person. As the group of patients with both TKR and THR was small, we did not include these in the final adjusted analysis. We considered a *P-*value of less than or equal to 0.05 to be significant, and all tests were two-tailed (SPSS version 15.0, SPSS Incorporated 2006, Chicago, IL, USA).

In Iceland a population-wide genealogical database has been compiled, which contains information about genealogical links between more than 95% of all Icelanders born since 1703. More than 715,000 individuals are registered in the database. This tool can therefore be used to look for aggregation of diseases and traits within families.

Due to the relatively small numbers in some work classes we merged groups in the genealogic analysis. Managers and professionals, technicians and clerks, service and shop workers and housewives were grouped together as light work. Craft workers and operators and unskilled labourers were grouped together as heavy labour. We then plotted all parent-child relationships that existed within our cohort and added the occupational status of the parent as a parameter. This enabled us to do a logistic regression analysis of the inheritance of occupation.

## Results

### Case and control characteristics

The mean age of cases, that is, subjects who had undergone TKR, THR or both for OA, was slightly higher than the age of controls. For men the BMI was slightly higher for cases than controls. Women who had undergone TKR or TKR and THR had higher BMI than controls, but women in the THR group did not (Table 2). For women, work classes were similarly distributed amongst cases and primary controls. However, for men this distribution was not even, with TKR and THR patients being overrepresented most notably among farmers.

**Table 2:** Characteristics of cases and controls

	THR only n = 896	TKR only n = 400	THR and TKR n = 112	Controls n = 1082
Sex, n (%)				
Men	399 (69.3)	134 (23.3)	43 (7.5)	490
Women	497 (59.7)	266 (32.0)	69 (8.3)	592
Age, mean (SD) years				
Men	74.7 (7.4)	73.9 (6.5)	74.2 (6.5)	71.0 (7.0)
Women	74.9 (7.8)	73.5 (7.2)	74.3 (8.1)	70.5 (7.1)
BMI, mean (SD) kg/m^2^				
Men	26.0 (3.2)	26.6 (3.2)	26.8 (3.1)	25.4 (2.9)
Women	25.2 (4.2)	27.0 (4.4)	26.3 (3.1)	25.2 (3.5)
Occupation, n (%)				
Managers and professionals				
Men	27 (6.8)	7 (5.2)	3 (7.0)	59 (12.0)
Women	46 (9.3)	15 (5.6)	6 (8.7)	48 (8.1)
Technicians and clerks				
Men	39 (9.8)	11 (8.2)	1 (2.3)	60 (12.2)
Women	67 (13.5)	27 (10.2)	7 (10.1)	95 (16.0)
Service and shop workers				
Men	28 (7.0)	5 (3.7)	3 (7.0)	30 (6.1)
Women	89 (17.9)	43 (16.2)	8 (11.6)	106 (17.9)
Farmers				
Men	140 (35.1)	43 (32.1)	10 (23.3)	83 (16.9)
Women	77 (15.5)	52 (19.5)	18 (26.1)	95 (16.0)
Fishermen				
Men	36 (9.0)	25 (18.7)	14 (32.6)	62 (12.7)
Women	0 (0)	0 (0)	0 (0)	0 (0)
Craft workers				
Men	78 (19.5)	30 (22.4)	6 (14.0)	122 (24.9)
Women	69 (13.9)	40 (15.0)	5 (7.2)	82 (13.9)
Operators and unskilled labour				
Men	51 (12.8)	13 (9.7)	6 (14.0)	74 (15.1)
Women	46 (9.3)	35 (13.2)	11 (15.9)	70 (11.8)
Housewives				
Men	0 (0)	0 (0)	0 (0)	0 (0)
Women	103 (20.7)	54 (20.3)	14 (20.3)	96 (16.2)

THR: total hip replacement; TKR: total knee replacement

### Recreational physical activity

In general our participants reported very low recreational physical activity. However, there were missing data for recreational activity in 20 to 26% of the questionnaires for different work classes. This was likely due to inadequately stated questions, and many of those who had not pursued recreational physical activity failed to answer the question. Farmers had the greatest proportion of non-responders for the question on recreational physical activity. We tested our logistic regression models with the addition of recreational physical activity as a covariate and the results presented remained essentially the same.

### Total knee or hip joint replacement for OA and occupation in men

The mean age of onset of knee symptoms for men who had TKR was 50.3 years. It was lowest among the technicians and clerks (45.0) and highest in the service and shop workers (61.8), but the difference was not statistically significant. For men with THR the mean age for onset of symptoms was 54.0 years, lowest among the fishermen (49.9) and highest in the service and shop workers (58.3). The difference was not statistically significant.

A sex-stratified multivariable logistic regression model with occupation as the exposure variable and case-control status as the dependent variable adjusted for age and BMI was done. For occupation we used managers and professionals as the reference group. We found a strong association for TKR in farmers with OR 5.1 (95% CI 2.1 to 12.4). Fishermen had an OR of 3.3 (95% CI 1.3 to 8.4), and craft workers OR 2.5 (95% CI 1.0 to 6.2). For THR in farmers we found an OR of 3.6 (95% CI 2.1 to 6.2). THR for service and shop workers also showed a significant association in this model with OR 2.1 (95% 1.0 to 4.1). Other work classes did not differ significantly from the reference group (Table 3).

**Table 3:** Odds ratios for different work classes for having a joint replacement

	Men		Women	
	TKR	THR	TKR	THR
Technicians and clerks	2.0 (0.71 to 5.7)	1.6 (0.85 to 3.0)	0.93 (0.44 to 2.0)	0.74 (0.44 to 1.3)
Service and shop workers	1.5 (0.41 to 5.2)	2.1 (1.0 to 4.2)	1.3 (0.63 to 2.6)	0.79 (0.48 to 1.3)
Farmers	5.1 (2.1 to 12.4)	3.6 (2.1 to 6.2)	1.4 (0.67 to 2.7)	0.62 (0.36 to 1.0)
Fishermen	3.3 (1.3 to 8.4)	1.3 (0.69 to 2.5)	None in group	
Craft workers	2.5 (1.0 to 6.2)	1.5 (0.87 to 2.7)	1.2 (0.59 to 2.5)	0.66 (0.39 to 1.1)
Operators and unskilled labour	1.4 (0.5 to 3.8)	1.4 (0.78 to 2.6)	1.4 (0.66 to 2.9)	0.60 (0.34 to 1.1)
Housewives	None in group		1.2 (0.58 to 2.4)	0.67 (0.40 to 1.1)

Numbers are odds ratio (95% confidence interval).

Managers and professionals are used as a reference group. THR: total hip replacement; TKR: total knee replacement

### Total knee or hip joint replacement for OA and occupation in women

The mean age of onset for knee symptoms in women who had TKR was 49.5. The differences between work classes were not statistically significant. For women with THR the mean age for onset of symptoms was 53.5 years, with no statistically significant differences between work classes. As with men we did a logistic regression model with the same parameters, finding no statistically significant difference between the work classes for TKR or THR (Table 3).

### Inheritance

Because our cohort was cross-sectional and the cases were 60 years of age or older, we had relatively few parent-child relationships. However, in the entire group (cases and controls combined) that remained before exclusion due to age (n = 5,386) we had several parent-child relations. By cross referencing our data with the genealogy database we were able to identify 474 men and 576 women whose father was also in our database and 629 men and 887 women whose mother was also in our database. By cross tabulating this we were able to determine how occupation was *inherited *to the next generation. We did this for all of our work class categories and calculated the odds ratio (OR) for *inheriting *the father's or the mother's work class. Several work classes had a significant OR for inheritance, farmers by far the greatest (Table 4).

**Table 4:** The odds ratios for inheriting parent's occupation class

	Father has same occupation	Mother has same occupation
Managers and professionals		
Men	3.1 (1.4 to 6.6)	2.4 (1.2 to 5.1)
Women	3.8 (2.0 to 7.3)	3.0 (1.9 to 5.0)
Technicians and clerks		
Men	2.3 (1.2 to 4.4)	1.6 (0.9 to 2.8)
Women	1.3 (0.8 to 2.2)	1.2 (0.8 to 1.8)
Service and shop workers		
Men	2.3 (0.6 to 8.2)	1.6 (0.9 to 2.8)
Women	1.7 (0.7 to 4.1)	1.0 (0.7 to 1.6)
Farmers		
Men	17.0 (7.7 to 37.7)	8.9 (5.0 to 15.8)
Women	17.5 (6.6 to 46.1)	11.4 (6.1 to 21.2)
Fishermen		
Men	5.7 (3.0 to 10.6)	N/A
Women	N/A	N/A
Craft workers		
Men	1.8 (1.1 to 2.9)	2.6 (1.5 to 4.3)
Women	0.7 (0.3 to 1.9)	2.2 (1.2 to 4.3)
Operators and unskilled labour		
Men	1.5 (0.7 to 3.1)	1.7 (0.9 to 3.2)
Women	0.8 (0.3 to 2.5)	1.8 (0.9 to 3.6)
Housewives		
Men	N/A	N/A
Women	N/A	2.1 (0.9 to 5.1)

Numbers are odds ratio (95% confidence interval). N/A: not applicable.

## Discussion

The purpose of this study was to explore whether occupation is associated with total knee or hip replacement for OA in Icelandic men and women.

Male farmers differed significantly from other work classes, having a greatly increased likelihood for both TKR and THR for OA. In the results presented in Table 3, we used managers and professionals as the reference group since we deemed that these had the lightest physical workload. It is possible to motivate the choice of other work classes as a reference group so we tested this model with each of the different work classes as a reference group. When exploring the association with TKR, we found a significant OR in some cases for other occupations than male farmers, but only the farmers differed consistently from the other work classes. For THR the results were similar. Male fishermen had a significant OR for TKR when using managers and professionals (OR 3.3, 95% CI 1.3 to 8.4) or operators and unskilled labourers (OR 2.4, 95% CI 1.1 to 5.2) as a reference group. Farmers had a consistently and significantly high OR regardless of reference group, except when using fishermen as a reference group. This suggests that male fishermen are associated with having TKR.

For women we found no differences between the work classes for TKR or THR. In this generation there was a gender difference in physical workload at the farm. The male farmer would be mostly responsible for the heavier work and the women would have a lighter workload, so the physical workload of the male farmer is not comparable to the workload of the female farmer.

There was no significant difference in mean age at onset of symptoms within the different work classes. If such a difference had existed it might lead to an overrepresentation of the work class that had lower mean age at symptom start.

We found a very strong inheritance for the farming occupation. Since few parent-child relationships were found within the cohort after individuals younger than 60 years were excluded, we calculated the odds ratio for how occupation is passed on from parent to child for the cohort prior to age exclusion. This confirmed that the farming profession is to a great extent passed on from parents to their children. Of the father-son relationships that we found in our cohort prior to exclusion due to age, 83% of farmers were sons of farmers. This might in some part explain the association between TKR/THR and farming. One possible explanation is that children, especially the sons, who were raised on farms, participated in heavy manual labour at a younger age than urban children would, and this might increase the prevalence of OA in this group. There is extensive familial clustering of OA in Iceland and OA cases can be traced to a relatively low number of founders (ancestors) compared to controls [33]. It is not unlikely that by chance these founders were farmers. Taking into account the strong inheritance of the farming profession, this might lead to an enrichment of genes associated with OA amongst the farmers of today. Because OA usually does not become evident until later age, it is not possible to truly determine the case or control status of the younger individuals. This limited our ability to establish the relative contribution of occupation and heredity for OA. A continued follow-up of this cohort may clarify this.

Previous studies on this topic have limitations, as does the present study. Some studies have been limited to a certain geographic area [8,9,11] and a selection bias might occur if persons in certain occupations had moved to another area after receiving their diagnosis, for example, because they needed to change occupation or for better health care. Our study was based on the entire population in Iceland and data were from all hospitals in the country.

In several previous investigations workload was defined from certain tasks [5,11] (for example, kneeling, squatting) rather than the occupational title. Participants in these studies were asked to grade how much they lifted, kneeled, squatted, and so on, during different periods of their life. In a study [34] that compared the correlation between self-reported and observed tasks at work, the coefficient of determination (r^2^) was as low as 0.15, meaning that in some of the cases 61% of study participants were not reporting correctly. If recall bias is added to this, the correlation is probably poorer. We chose to base our classification on job title which should be less prone to recall bias. However, our approach does not take into account the differences between different occupations that are classified into the same group or the differences that can be present within a given job title. Farming in Iceland has been very homogenous, all being single family farms with cattle, sheep or a mixture of both. Fishermen can also be considered a homogenous work class. Other work groups presented here are more heterogeneous as we had several subclasses combined in, for example, service and shop workers.

The definition of OA varies between different studies. Some studies define OA by radiological findings, with or without clinical symptoms, while others use joint replacement as definition. OA varies in its severity and radiological findings have poor correlation to the clinical presentation [18,19]. Total joint replacement is generally done for patients with severe OA symptoms not managed satisfactorily by other interventions. In contrast to, for example, a definition based on radiographs only, a case definition based on joint replacement represents a significant disease burden. A limitation of this case definition is, however, the multiple influences beyond symptoms on the patient and health professional decision of joint replacement [35]. A further limitation with our choice of case definition was that we surely had some false negatives in our control group, that is, some of the controls might after the end of the study develop OA that requires joint replacement. This would lead to a bias towards the null. To try to minimise this we chose to include only individuals that were 60 years of age or older at study entry. Because we used joint replacement as a definition for our cases, persons that were deemed too ill to have joint replacement are also classified as controls, even though their disease severity motivates that they should be classified as cases. This healthy patient selection bias is also towards the null.

An additional confounder is that persons with physically demanding occupations could be more at risk for joint trauma and develop their OA at a younger age [9,36]. They might also experience greater problems with performing their work after the onset of OA and thus seek help earlier and be overrepresented as cases. We found no statistical difference in the age of onset of symptoms between the different work classes so this does not seem to be a problem in the current study. However, we cannot exclude the possibility of a healthy worker effect where only the most healthy survive within the trade (that is, young individuals with, for example, hip pain would be forced into another trade than farming or fishing). This would also lead to bias toward the null. It has been suggested that farmers are less willing to seek healthcare for musculoskeletal problems [37], which would also lead to a bias toward the null.

Our analysis of recreational physical activity was limited by the fact that 25% of, the participants did not answer this question. No other question in our questionnaire had such a high percentage of missing values. We suggest that the most probable reason is that it was not clearly enough stated that those that had not been active in any recreation physical activity should state that fact. We nevertheless tested different models to compensate for our missing values. None had any significant impact. It is not surprising from the biological standpoint that the effects of recreational activity a few hours per week should be much less than the effects of the occupation eight hours per day, five days per week and in some cases (for example, farmers) much more than that.

Changes in access to joint replacements over time might be a possible confounder. More than 90% of our controls were born after 1920 and joint replacement had become readily available in Iceland after 1985, so we do not believe that many of our controls were in fact individuals in need of arthroplasty that were unable to have an operation due to poor availability.

The controls were not drawn from the source population which the cases were derived from, but were relatives of the cases. This was dictated by the fact that the current study is a part of a larger project studying the effect of genes and environment on the risk of OA in the Icelandic population. Any bias introduced by the control selection (first degree relatives), would likely be bias towards the null because it is more likely to find the same profession also within immediate family members than from the background population, that is, yielding an increased frequency of, for example, farmers or fishermen also in the control sample.

The association with hip osteoarthritis amongst male farmers has been established in several studies [7-9,13,22,23], including one prospective study [23]. One previously published study suggested an association between knee OA and farming [12], while another found no such association [38], although that study has not been ranked as being of high quality. To the best of our knowledge this is the first population-based study to suggest an association with both TKR and THR and farming in the same sample. Few studies have been done on female farmers.

The evidence for other professions is less compelling [11,13]. We found no significant association for other professions, other than an increased likelihood for TKR due to knee OA for fishermen, when compared to more sedentary occupations. We found that for male service and shop workers with THR and male craft workers with TKR, the OR were borderline significant and in a larger sample these associations might reach statistical significance. For women, no OR was statistically significant, but for THR they were all less than 1. This might indicate that the reference group (managers and professionals) has an increased risk for THR in women.

## Conclusions

In this study we found that male farmers have an increased likelihood for both knee and hip joint replacement due to OA. We have also shown that farming is the occupation that is most commonly passed on within families, that is, most farmers have parents who are farmers. Thus genes associated with OA inherited among farmers could interact with the workload-associated risk due to farming.

## Abbreviations

BMI: body mass index; CI: confidence interval; ILO: The International Labour Organization; OA: osteoarthritis; OR: odds ratio; THR: total hip replacement; TKR: total knee replacement

## Competing interests

The authors declare that they have no competing interests.

## Authors' contributions

JF collected the data and was responsible for data analysis and drafting the manuscript. JF, TI and SL conceived the study. TI, ME and SL revised the manuscript. All authors participated in data analysis and all authors approved the final version of the manuscript.
